# Main genetic factors associated with inflammatory bowel diseases and their consequences on intestinal permeability: involvement in gut inflammation

**DOI:** 10.1007/s00535-025-02289-x

**Published:** 2025-08-21

**Authors:** Arthur Mauduit, Emmanuel Mas, Nuria Solà-Tapias, Sandrine Ménard, Frédérick Barreau

**Affiliations:** 1 INSERM UMR 1220, INRAE UMR 1416, ENVT, Université de Toulouse Paul Sabatier, 31000 Toulouse, France; 2 Service de Gastroentérologie, Hépatologie, Nutrition, Diabétologie Et Maladies Héréditaires du Métabolisme, Hôpital Des Enfants, CHU de Toulouse, 31300 Toulouse, France; 3 INSERM UMR1220—Institut de Recherche en Santé Digestive (IRSD)-Purpan Hospital, BP 3028, 31024 Toulouse Cedex 03, France

**Keywords:** Crohn’s disease, Ulcerative colitis, Nod2, Autophagy, Endoplasmic reticulum stress, Th-17, Tight junctions

## Abstract

Crohn’s disease (CD) and ulcerative colitis (UC), the two main subtypes of inflammatory bowel diseases (IBD), are chronic relapsing inflammatory disorders of the gastrointestinal tract. IBD are multifactorial diseases with a complex etiology, involving an intricate interaction between environmental and genetic factors. Since the discovery of *NOD2* gene in 2001, genome-wide association studies have reported more than 200 IBD susceptibility loci. The strongest associations highlighted five main pathways as altered in IBD: bacterial sensing (*NOD2*), autophagy (*ATG16L1*, *IRGM…)*, endoplasmic reticulum stress (*XBP1*, *ARG2…*), Th-17 immune pathway (IL23-receptor), and the vitamin D receptors (VDR). The pathophysiology of IBD results from an abnormal immune response toward an altered gut microbiota. Although the *primum movens* remains unknown, an increased intestinal permeability is clearly involved in the genesis of this abnormal crosstalk, leading to whole tissue inflammation. Thus, an excessive intestinal permeability, or “leaky gut”, has been described to precede the development of CD. Moreover, in IBD, intestinal permeability is described to be a sensitive prognostic indicator of relapse in patients with quiescent IBD. Thus, the aim of this review is to highlight the molecular and cellular mechanisms by which the main pathways associated with IBD could contribute to alter the intestinal permeability to favour and/or exacerbate chronic inflammation, leading to debilitating diseases.

## Introduction

Crohn’s disease (CD) and ulcerative colitis (UC), the two main subtypes of inflammatory bowel disease (IBD), are chronic relapsing inflammatory disorders of the gastrointestinal tract [1]. Patients suffer from relapsing flares with diarrhea, abdominal pain and rectal bleeding. IBD can be complicated by strictures, fistula, perforations of the intestine and cancers [1]. Although the etiology of IBD is poorly understood, many data suggest that inflammatory lesions could be driven by environmental stimuli, inducing an immune dysfunction in genetically predisposed individuals [1]. Since the discovery of *NOD2* gene in 2001 [2], genome-wide association studies (GWAS) have reported more than 200 IBD susceptibility loci [3]. The strongest associations highlighted five main pathways as altered in IBD: bacterial sensing (*NOD2*), autophagy (*ATG16L1*, *IRGM…)*, endoplasmic reticulum (ER) stress (*XBP1*, *ARG2…*), Th-17 immune pathway (IL23-receptor), and the vitamin D receptors (VDR) (Table 1) [3–5].

**Table 1:** Genetic associations with Crohn disease, ulcerative colitis and inflammatory bowel diseases

	Crohn disease	Ulcerative colitis	IBD
Nucleotide-binding oligomerization domain 2	Associated	Not associated	Not associated
Endoplasmic reticulum stress (Agr2, XBP1)	Associated	Not associated	Associated
Autophagy (ATG16l1, IRGM, ULK1 and LRRK2)	Associated	Not associated	Not associated
IL-23 receptor	Associated	Associated	Associated
Vitamin D receptor	Not associated	Not associated	Associated

The intestinal epithelium, which separates the luminal content from the mucosal immune system, is an important defensive line to protect the host from the luminal content (bacterial and food antigens, toxins, etc.…) and minimize gut inflammatory responses [6]. The continuous exposure of the intestinal tissue to microorganisms keeps the mucosa in a state of physiological inflammation, which balances tolerogenic and inflammatory type responses to maintain homeostasis. Today, IBD pathophysiology is thought to result from an excessive immune response towards an altered gut microbiota. Although the *primum movens* remains unknown, an increased intestinal permeability (IP) is clearly involved in the genesis of this abnormal crosstalk, leading to whole tissue inflammation [7].

In IBD, IP is described to be a sensitive prognostic indicator of relapse in patients with quiescent disease [8, 9]. Thus, quiescent CD patients with an elevated IP exhibited an increased risk of relapse compared to a quiescent CD with a reduced IP [8, 9]. Moreover, an excessive IP has been also described to precede the development of CD [10–12]. Thus, the aim of this review is to highlight the molecular and cellular mechanisms by which the main pathways associated with IBD (NOD2, autophagy, ER stress and Th-17 immune pathway) could contribute to IP alterations.

## Intestinal permeability

IP represents one of the actors of intestinal barrier and contributes to exchanges between mucosal and serosal compartments of epithelium. The definition of IP is “the facility by which the intestinal epithelium allows molecules to pass through by non-mediated passive diffusion” [13]. Intestinal passage, in a more general manner, is measured by unidirectional fluxes (expressed in mol.h^−1^.cm^−2^ or cm/s) (for review [14]). IP, inflammation and gut microbiota are regulating themselves. Gut physiology is dependent on an appropriate relationship between those 3 actors of the intestinal barrier. In complex diseases like IBD associated with inflammation and microbiota dysbiosis deciphering the *primun movens* is difficult. To get a better view, the aim of this review is to describe the links between genetic factors associated with IBD and their ability to impair signaling pathways regulating IP.

**Fig. 1:**
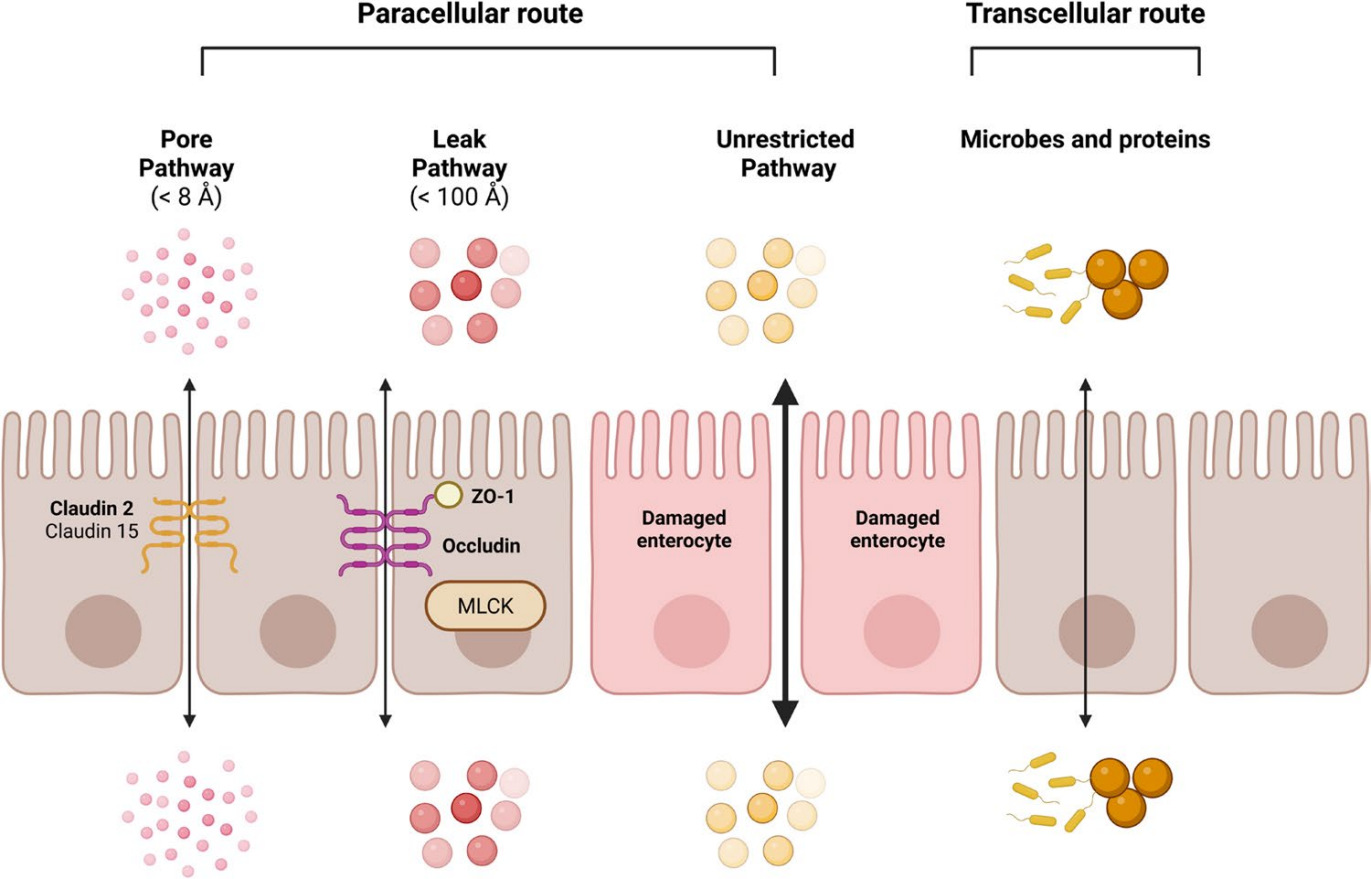
The different pathways involved in intestinal permeability. Intestinal permeability is divided into the paracellular route and the transcellular route. The paracellular route includes the “pore” pathway (mainly regulated by claudins, e.g. Claudin 2 and Claudin 15), the “leak” pathway (mainly regulated by Occludin, Zonula Occludens 1(ZO-1)) and Myosin Light Chain Kinase (MLCK) and the “unrestricted” pathway formed by the passage between damaged cells. Larger particles like microbes and proteins use the transcellular route

Two main pathways have been identified: paracellular pathway for small molecules and transcellular pathway for large ones (Fig. 1). As previously stated, both para- and trans-cellular permeability are increased in IBD, exposing gut associated immune system to larger amounts of luminal antigens. Para- and trans-cellular pathway functions and mechanisms have been extensively described in healthy individuals and IBD patients (for reviews [15, 16]). We will present here a brief summary.

### Paracellular permeability

Paracellular permeability allows the passage of water, solutes and ions and is determined by the size of tight junction (TJ) pores (Fig. 1) [17]. Four transmembrane families of proteins (occludin, claudin, junctional adhesion molecule and tricellulin) contribute to TJ formation [17]. The intracellular domains of these transmembrane proteins interact with cytosolic scaffolding proteins (zonula occludens proteins), which in turn anchor the transmembrane proteins to the peri-junctional actin-myosin meshwork [17]. There are two distinct routes across TJ of normal epithelium termed “pore” and “leak” pathways (Fig. 1). Moreover, a paracellular last route named “unrestricted pathway” has been described when intestinal homeostasis is disrupted, e.g. when the intestinal epithelium is severely damaged (Fig. 1). The pore permeability presents a high-capacity, size- and charge-selective route, whereas the leak pathway exhibits a low-capacity pathway that has limited selectivity. Pore permeability is mainly regulated by the subset of claudins, while leak permeability is more regulated by zonula occludens type 1 (ZO-1), occludin, and myosin light chain kinase (MLCK) (Table 2) [18]. The circumferential contraction of the actinomyosin ring triggering the opening of the TJ is regulated by the MLCK [17, 19]. Other mechanisms like endocytosis of TJ proteins, regulation of TJ genes’ expression and cell apoptosis (unrestricted pathway) regulate the paracellular route. In CD and UC, increase of paracellular permeability is accompanied by abnormal expression and composition of TJ proteins (Table 3) [20].

**Table 2:** Molecular mechanisms involved in paracellular and transcellular permeability

	Paracellular pathway		Transcellular pathway
	Pore pathway (< 8 A)	Leaky pathway (< 100 A)	
Myosin Light Chain Kinase	Not involved	Involved; ↑	Involved; ↑
Occludin	Not involved	Involved; ↓	Not involved
Claudin 2	Involved; ↑	Involved; ↑	Not involved
Claudin 4	Involved by antagonizing Claudin 2; ↓	Not involved	Not involved
Claudin 5	Unclear Evidence	Involved; ↓	Not involved
Claudin 15	Involved;	Not involved	Not involved
Zonula occludens 1	Not involved	Involved; ↓	Not involved

**Table 3:** Molecular mechanisms involved in increased permeability in Crohn disease and ulcerative colitis

	Paracellular permeability		Transcellular permeability	
	Pore	Leaky	Proteins	Microbes
Crohn disease	Increased	Increased	Increased	Increased
MLCK	Not involved	Involved	Involved	Involved
Claudin 2	Increased	Increased	Not involved	Not involved
Occludin	Not involved	Decreased	Not involved	Not involved
Claudin 4	Decreased	No evidence	Not involved	Not involved
Claudin 15	Decreased	Not involved	Not involved	Not involved
Zonula occludens 1	Not involved	Decreased	Not involved	Not involved
Ulcerative colitis	Increased	Increased	Increased	Not Increased
MLCK	Not involved	Increased	Not involved	Not involved
Claudin 2	Increased	Increased	Not involved	Not involved
Occludin	Not involved	Decreased	Not involved	Not involved
Claudin 4	Decreased	No evidence	Not involved	Not involved
Claudin 15	Decreased	Not involved	Not involved	Not involved
Zonula occludens 1	Not involved	Decreased	Not involved	Not Involved

### Transcellular permeability

Large particles use the transcellular route (Fig. 1) [19]. Although epithelial and dendritic cells are able to capture antigens and microbes, the transcellular transport is mainly ascribed to M-cells overlying isolated lymphoid follicle or Peyer’s patches [21]. The transcellular route across M-cells and other epithelial cells exhibits two classical pathways depending on the particle’s size. Proteins and bacterial products can be taken up by endocytosis [22]. Larger particles (bacteria…) are captured via macropinocytosis or phagocytosis [22]. In case of epithelium disruption, enhanced and non-controlled passage of antigens and microbes could lead to pathological conditions such as chronic inflammation and carcinogenesis [17]. Increased transcellular permeability to large protein model (horseradish peroxidase, HRP) is observed in both CD and UC [23], whereas transcellular permeability to *E. coli* is only increased in CD (Table 3) [24]. Of note, receptor mediated transport of luminal antigen also belong to the transcellular pathway but to our knowledge it has not been identified to be relevant in IBD.

It is noteworthy that various methods have been developed regarding IP measurement but they do not provide the same information regarding IP per se.[25] Everyone has to keep a sense of criticism when concluding on IP. Briefly, three main methods have been described: in vivo measurement by oral probes, ex vivo measurement in Ussing chamber, and detection of biomarkers of feces in blood. All these methods and the choice of probes have advantages and limitations that are well discussed in this review [26].

## Intestinal permeability and IBD

It is now clear that a defect in IP precedes the development of inflammation in CD. This is now well accepted since large-scale longitudinal studies of first-degree relatives have demonstrated that increased IP precedes clinical disease development by several years. [11] Moreover, old and recent clinical studies have clearly reported that elevated paracellular permeability is a risk factor for CD relapses [8, 27]. For UC, there is nowadays no evidence supporting that IP increases before the development of the disease as well as elevated paracellular permeability is a risk factor for UC relapse. Thus, in UC patients, increased permeability is probably the result of ongoing inflammation and mucosal damage. In contrast, in CD, the presence of anti-Saccharomyces cerevisiae antibodies (ASCA) is well-established. ASCA positivity is associated with CD and is thought to be, at least in part, a consequence of increased IP, which allows greater exposure of the immune system to luminal antigens [28]. Nevertheless, in these diseases, elevated IP also contributes to perpetuating the chronic inflammation by allowing luminal antigens to access the immune system.

Inflammation in CD can develop in any part of the gastrointestinal tract, from the mouth to the anus, but most commonly involves the terminal ileum and the colon while UC develops only in the rectum and the colon. This phenomenon changes the way how permeability is impacted in both diseases.

Concerning the transcellular permeability, although the elevated passage of proteins in both CD and UC, an increased bacterial translocation has been described only in CD, not in UC patient [23, 24]. In genetic mouse model of CD (*Nod2*^*KO*^ mice), the excessive bacterial translocation observed across the Peyer’s patches (PP) of *Nod2*^*ko*^ mice is mediated by MLCK, since its inhibition by ML-7 treatment suppressed this bacterial translocation (Tables 2 and 3) [29].

**Fig. 2:**
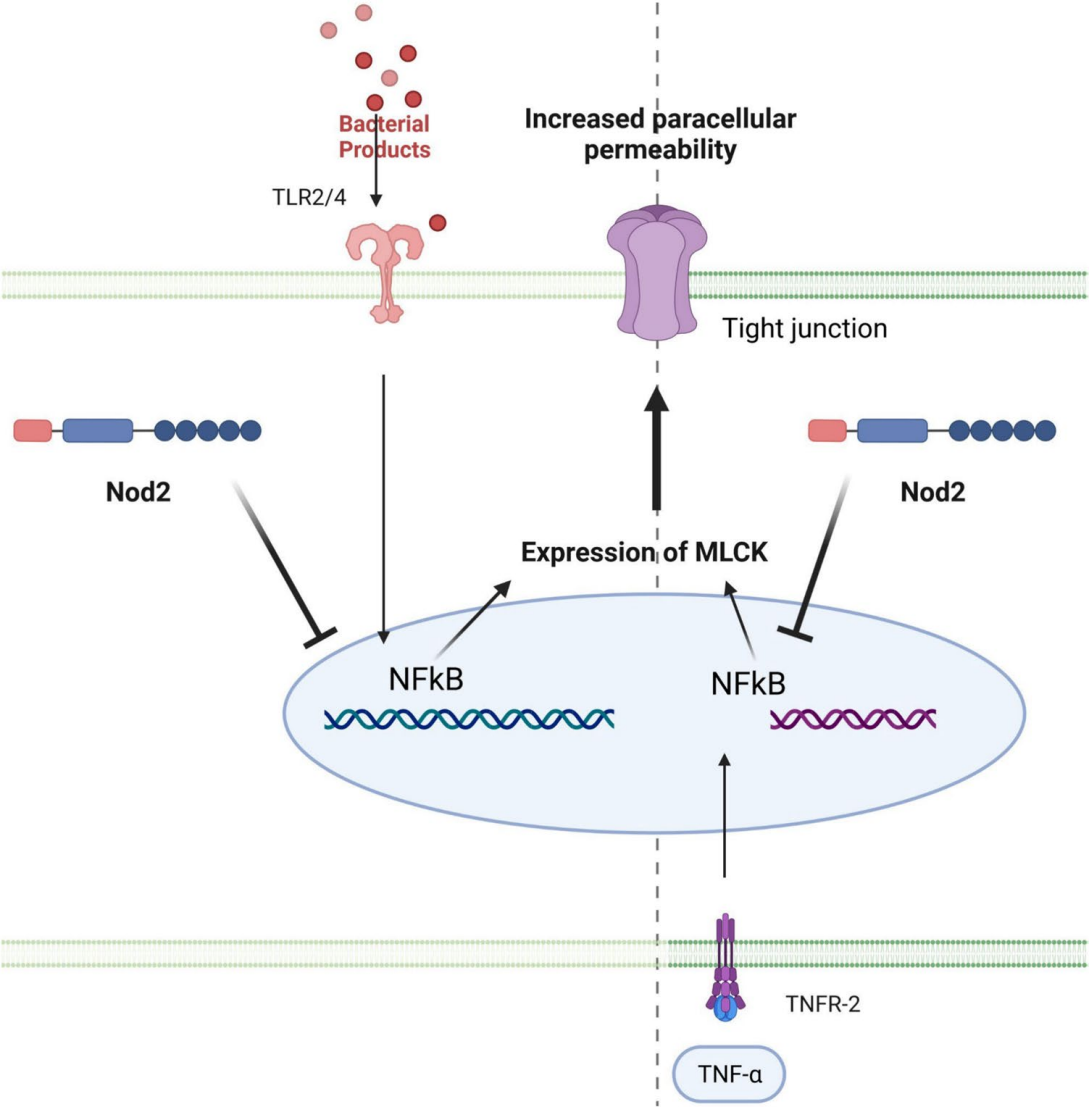
The mechanisms by which Nod2 maintains the intestinal permeability. In context of TLR activation and cytokines stimulation, Nod2 regulates the intestinal permeability. Thus, TLR2/4 activation increases the intestinal permeability by increasing the MLCK expression and activity. This increased expression and activity of the MLCK triggered by the nuclear translocation of NF-κB is suppressed by Nod2 stimulation by MDP. Similarly, Nod2 stimulation is also able to supress the increased intestinal permeability induced by TNF-α. *MLCK* Myosin Light Chain Kinase, *Nod2* nucleotide-binding oligomerization domain 2, *NF-κB* Nuclear factor-kappa B, *TLR* Toll like receptors, *TNF-α* Tumor Necrosis Factor α, *TNFR-2* Tumor Necrosis Factor receptor 2

With regard to paracellular permeability, an increase has been reported in both CD and UC but the underlying mechanisms are different (Tables 2 and 3). In CD, the pore pathway is slightly implicated, but the main pathways involved are the leak pathway and the ‘unrestricted pathway’ (Fig. 1 and Table 3). In the leak pathway, MLCK is strongly involved, as is the loss of TJ sealing proteins (Occludin, Claudin 5 and Claudin 8), compared to UC.[18]. MLCK expression and activity in CD is directly enhanced by inflammatory molecules such as TNF-α. TNF-α upregulates MLCK through NF-κB (Fig. 2, left panel). In UC, the pore pathway is the main driver of paracellular permeability regulation (Table 3). Claudin 2 is an important regulator of IP in both diseases, but its expression is increased more markedly in active UC than in active CD, suggesting that it may play a significant role in increasing IP, particularly in UC (Tables 2 and 3) [20]. Claudin 2 expression is involved in the IL-13 signalling pathway, while TNF-α increases Claudin 2 expression in CD [30].

Finally, several recent clinical studies have evidenced that some molecular partners (Occludin, Claudin 2) involved in the increased paracellular permeability can have utility in the diagnosis of both UC and CD [31, 32]. Additionally, Claudin-2 may be helpful in evaluating the intensity of the inflammatory process [31]. Thus, Claudin-2 appeared to be a very sensitive marker of disease activity in IBD and correlated with the severity of UC [32]. Anti-TNF-α treatment improved the value of occluding and claudin-2, indicating its beneficial effect on the integrity of TJ in UC [31, 33]. About the MLCK, one old paper has evidenced that MLCK protein expression and enzyme activity were increased in the intestinal epithelium according to the CD’s severity [34]. Nevertheless, concerning the expressions of ZO-1 and the disease severity, no study is today available.

## The genetic factors associated with IBD and their impacts on IP

### Nucleotide-binding oligomerization domain 2 (NOD2)

The *NOD2* gene is a member of the evolutionarily conserved Nod-like receptors family, which senses the muramyl dipeptide (MDP), which arises from the peptidoglycan degradation of the bacteria wall [35, 36]. In the past two decades, studies have reported that Nod2 plays a pivotal role in the regulation of chronic inflammatory conditions [37, 38]. *NOD2* polymorphisms were found to be associated with an increased risk of CD (Table 1). In the gut, Nod2 is expressed by both hematopoietic and non-hematopoietic cells forming the intestinal epithelium [38]. Upon ligand sensing, Nod2 undergoes oligomerization with Nod1 and/or other Nod2 receptors to form a signaling complex named *nodosome*.[39, 40] As a result, the formation of the nodosome promotes host defense through the production of cytokines, chemokines [41, 42], antimicrobial peptides (AMP) [43], and mucins [43, 44].

Although the mechanisms by which *NOD2* mutations influence the development of CD are poorly understood, CD-associated *NOD2* polymorphisms lead to a loss of function of MDP-downstream pathways, promoting excessive stimulation of immune and epithelial cells by altered microbiota to drive inflammation [38, 45, 46]. Thus, in the absence of functional NOD2 protein, TLR-2 and -4 are not negatively regulated and this leads to an excessive NF-κB response and production of inflammatory cytokines by immune cells [47, 48]. Similarly, in intestinal epithelial cells (IEC), the absence of functional NOD2 protein is associated with a reduced capacity to produce AMP and mucins [43, 44]. Finally, a functional Nod2 is also required to keep the intestinal stem cells in physiological condition allowing to the epithelium renewal [49]. In addition to its specific role in each epithelial and immune cells type, an abundant literature have reported the main role played by Nod2 in the regulation of IP in Humans as well as in animal models [37, 38, 50–55]. In Humans, *NOD2* polymorphisms are associated with an elevated paracellular permeability in both CD patients and their first-degree relatives [50–52]. Similarly, mice deleted or mutated for *Nod2* exhibited an increased para- and trans-cellular permeability [29, 43, 53, 54, 56]. These excessive para- and trans-cellular permeability, in *Nod2* deficiency mice, are due to a MLCK over-activation [29, 54].

In the context of TLR activation, Nod2 has also been described to regulate IP and the expression of cytokines (Fig. 2). Indeed, *Nod2* deficiency in mice is associated with excessive lymphoid tissue, increased expression of TLR2 and TLR4, CD4^+^ T cells, TNFα and IFNγ, and permeability in PP [29]. ML-7, a MLCK inhibitor, treatment reversed all those features in *Nod2*^−/−^ mice (Fig. 2). Nod2 appears as a regulator of TLR-2 and -4 activation pathway and protector of intestinal barrier. Furthermore, *Nod2* is expressed in hematopoietic and non-hematopoietic cells and both contribute to IP [43]. Nod2 deficiency in hematopoietic cells increased TNF-α and IFNγ secretion by CD4^+^ T cells triggering MLCK activation and intestinal hyperpermeability (Fig. 2).[56] In addition, NOD2 stimulation of non-hematopoietic cells by MDP decreased pro-inflammatory secretion by CD4^+^ T cells and restore normal IP [56]. In conclusion, Nod2 expression in hematopoietic and non-hematopoietic cells prevent intestinal hyper-permeability.

Interestingly, *NOD2* mutations also facilitate receptor mediated transcytosis of immune complexes. Indeed, *NOD2* mutations (loss of function) increases the expression of Dectin1 and Siglec5 on M cells allowing entry of IgA/STm immune complexes in human PP and mouse models [57]. This result is of particular interest since *Salmonella* gastroenteritis [58] as well as *Yersinia pseudotuberculosis* ileitis[59] increased the risk to develop CD [59].

### Endoplasmic reticulum stress

The ER is an essential subcellular organelle responsible for the synthesis and maturation (folding) of proteins, via chaperone and oxidation processes. Misfolded proteins accumulating in ER lead to ER stress. ER stress activates the cytoprotective unfolded protein response (UPR) to restore homeostasis. At physiological state, the chaperone GRP78 binds the three sensors of ER stress, i.e., protein kinase RNA (PKR)-like ER kinase (PERK), activating transcription factor 6 (ATF6), and inositol-requiring protein 1(IRE1), and thus inhibiting UPR. IRE1 detects an imbalance between misfolded proteins and chaperones, inducing unconventional splicing of X-Box Binding Protein 1 (XBP1) mRNA, and promoting cytoplasmic RNase and expression of various ER quality control genes. Anterior Gradient 2 (AGR2) overexpression is induced by UPR participating to ER homeostasis.

IBD genetic studies have identified susceptible risk alleles, such as *XBP1* and *ARG2*, both involved in ER-stress and UPR signalling (Table 1) [60]. Thus genetic deletion of components involved in UPR are linked with spontaneous gut inflammation and/or enhanced sensitivity to colitis [61]. Mice with IEC-specific deletion of *XBP1* exhibit a spontaneous development of gut inflammation carrying the hallmarks of human IBD including loss of Paneth cells, immune cells infiltration and ulcerations in the small bowel without modification of IP measured in vivo with FD4 [61]. IEC of the small intestine of Xbp1-KO mice present an increased GRP78, leading to enhanced apoptosis, a reduced number of Paneth cells and the absence of its secretory granules, together with a decreased release of AMP. Thus, dysregulation of ER-stress contributes to spontaneous inflammation in the small intestine, and compromises host defence against enteropathogens [62, 63]. Another study showed that Inositol-Requiring Enzyme 1β (Ire1*β*)^−/−^ mice exhibited increased ER stress and early exacerbated inflammation upon DSS colitis [64]. The development of spontaneous colitis is observed in mice deficient for Ire1α^−/−^ in IEC along with diminished number of goblet cells and increased IP measured in vivo with 4 kd Fitc-Dextran (FD). ER-stress signals showed downregulation of *XBP1*s mRNA and upregulation of CCAAT-enhancer-binding protein homologous protein (CHOP) known to induce apoptosis [65]. CHOP is also described to exacerbate the development of colitis by contributing to colonocyte apoptosis. Thus down-regulation of CHOP might contribute to ameliorate colitis [66]. Agr2 is a protein disulfide isomerase that plays an important role in gut homeostasis regulation. Agr2^−/−^ mice display abnormal location of Paneth cells into the ileal villi impairing the secretion of its proteins, a decreased number of goblet cells and a constitutive induction of ER stress in gut mucosa [67]. To our knowledge no measurement of IP have been performed in Agr2^−/−^ mice so far.

Basal levels of ER-stress are always present in the gut, but when cells are not able to properly manage this stress, the intestine undergoes through spontaneous inflammation and IEC are more sensitive to environmental factors triggering inflammation. Previous studies reported that colonic IEC from UC patients have a dysregulated activation of Eukaryotic Translation Initiation Factor 2 A (eIF2a) leading to changes in protein translation, including junctional proteins and secretory pathways, and altered colonic mucosa barrier function [68]. Ravindran et al. have demonstrated PERK protein upregulation in inflamed colonic tissue of UC and CD patients, compared to healthy controls [69]. Finally, in IBD and specifically in CD, the levels of AGR2 dimerization are deregulated, and this correlates with disease severity [70].

**Fig. 3:**
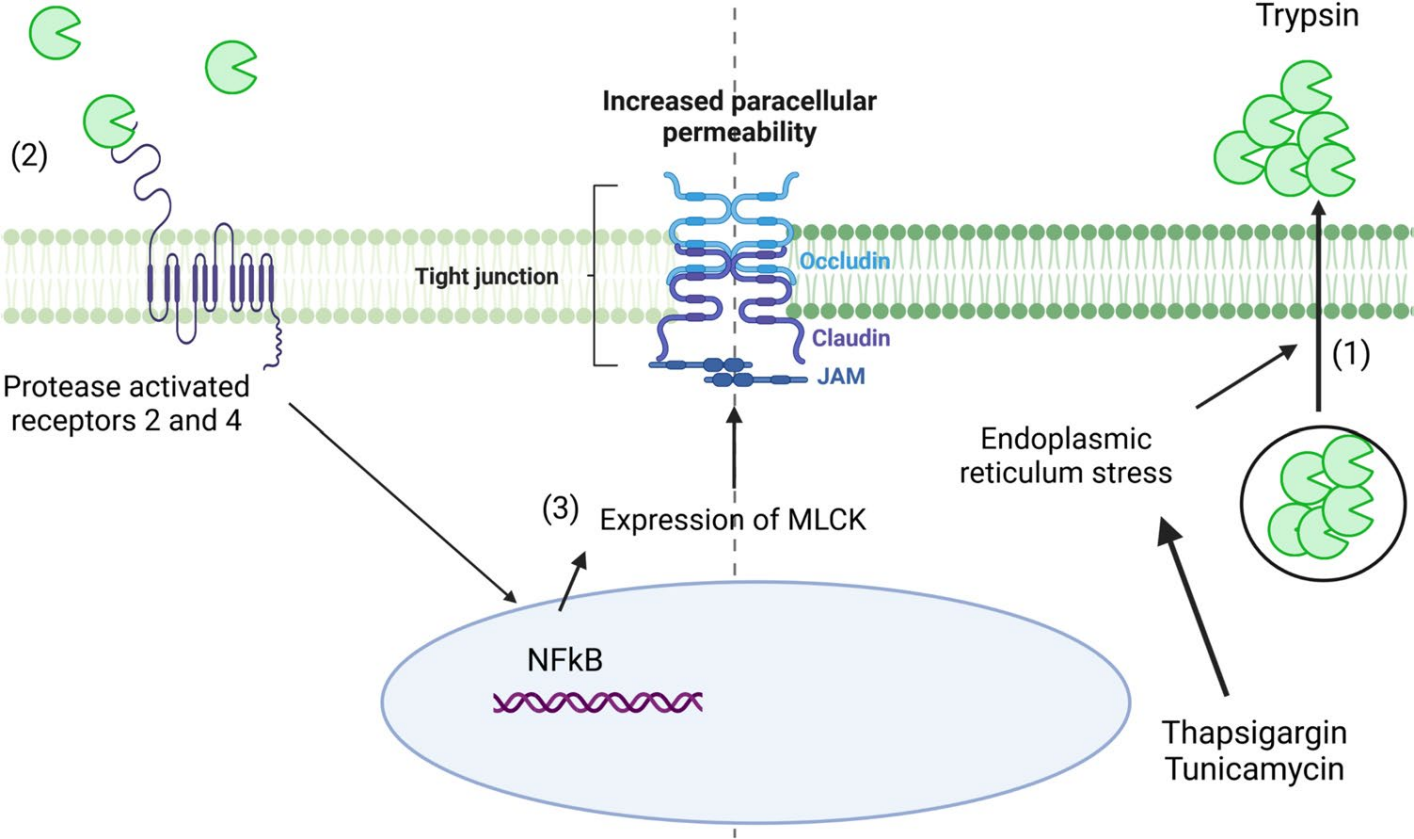
Mechanisms involved in the altered intestinal permeability induced by the endoplasmic reticulum stress. The induction of ER stress has been shown to increase paracellular permeability. This increased permeability is due to the release of trypsin proteolytic activity at the apical surface (1), which is induced by ER stress and activates the protease-activated receptors 2 and 4 (2). The activated PAR2 and PAR4 upregulate NFκB which stimulates the expression and activity of MLCK (3), thereby increasing paracellular permeability. *MLCK* Myosin Light Chain Kinase, *NF-κB* Nuclear factor-kappa B

Although the role of ER stress in inflammatory process has been extensively described, its role in IP has been only reported in few recent studies. Thus, Tunicamycin-induced ER stress reduced the trans-epithelial electrical resistance (TEER) of Caco-2 monolayers (Fig. 2).[71] This epithelial barrier dysfunction was not caused by caspase- or RIPK1-dependent cell death but was accompanied by lysosomal rupture [71]. Similarly, induction of ER stress by Tunicamycin or Thapsigargin increased the permeability to FD4 in rat colonic tissues mounted in Ussing chamber (Fig. 3).[72] More recently, we have shown that the increased permeability of Caco-2 monolayers stimulated by Thapsiargin is due to an excessive apically release of trypsin proteolytic activity (Fig. 3) [73]. Although a direct role of these proteases to break the TJ is one possibility, we have shown that proteases alter the permeability by activating protease activated receptors 2 and 4, leading to an activation of the MLCK.[73] Indeed, treated Caco-2 monolayers with ML-7, an inhibitor of MLCK, suppressed the increased permeability induced by Thapsigargin (Fig. 3).[73] To conclude, neither *XBP1* nor *AGR2* mutations alone affect IP as tested in mice models. However, it is worthwhile to mention that IP in Xbp1-KO mice have been tested in vivo and would need to be further addressed ex vivo with markers of various molecular weight.

### Autophagy

Autophagy is regarded as a vital metabolic process, degrading and recycling long-lived or misfolded proteins and useless organelles under physiologic and non-physiologic conditions. Since the initial reports in the mid-1950s, autophagy has been reported to be involved in the pathogenesis and progression of IBD. In IBD, autophagy has been widely revealed to regulate the onset and development of IBD via immune and inflammatory modulation [74, 75]. GWAS have identified variants in autophagy genes such as Autophagy related 16 like gene (*ATG16L*) and immunity-related GTPase family M (*IRGM*) as genetic risk factors for CD [74, 75]. GWAS approaches have identified a threonine-to-alanine substitution (T300A) in *ATG16L1* associated with an increased risk of developing CD but not UC. CD patients, homozygous for *ATG16L1/T300A* variant, present a “loss-of-function” of autophagy due to the impairment of autophagosome formation, which, in turn, displays an altered capacity to handle and clear cytoplasmic content such as bacteria like *Salmonella* or adherent-invasive *Escherichia coli* (AIEC) [76, 77]. In hypomorphic mice *ATG16L1/T300A risk allele* (ATG16L1^HM^ mice), Paneth cells exhibited abnormalities in their granules of secretion containing AMP and increased expression of inflammatory mediators [78]. In this study, homozygous CD patients for the *ATG16L1* risk variant display similar impaired structure in Paneth cells than those observed in ATG16L1^HM^ mice. Also, impaired autophagy is associated with nucleation and elongation of autophagosome in goblet cells, driving a deficiency in mucus secretion [79]. Moreover, physical interaction between NOD2 and ATG16L1 is described to play a pivotal role in the elimination of intracellular pathogens [80]. Thus, NOD2 recruits ATG16L1 and co-localize at the plasma membrane to facilitate the initiation of the autophagosome and the elimination of intracellular pathogens [80]. Finally, malfunction of NOD2 impacts not only the killing and handling of pathogenic microbes but also their presentation by the major histocompatibility complex class II (MHC-II) to induce immune response [81]. A single nucleotide polymorphism exhibits a decreased expression of IRGM and decreased capacity of autophagy to clear up intracellular bacteria leading to infection in CD patients [82].

**Fig. 4:**
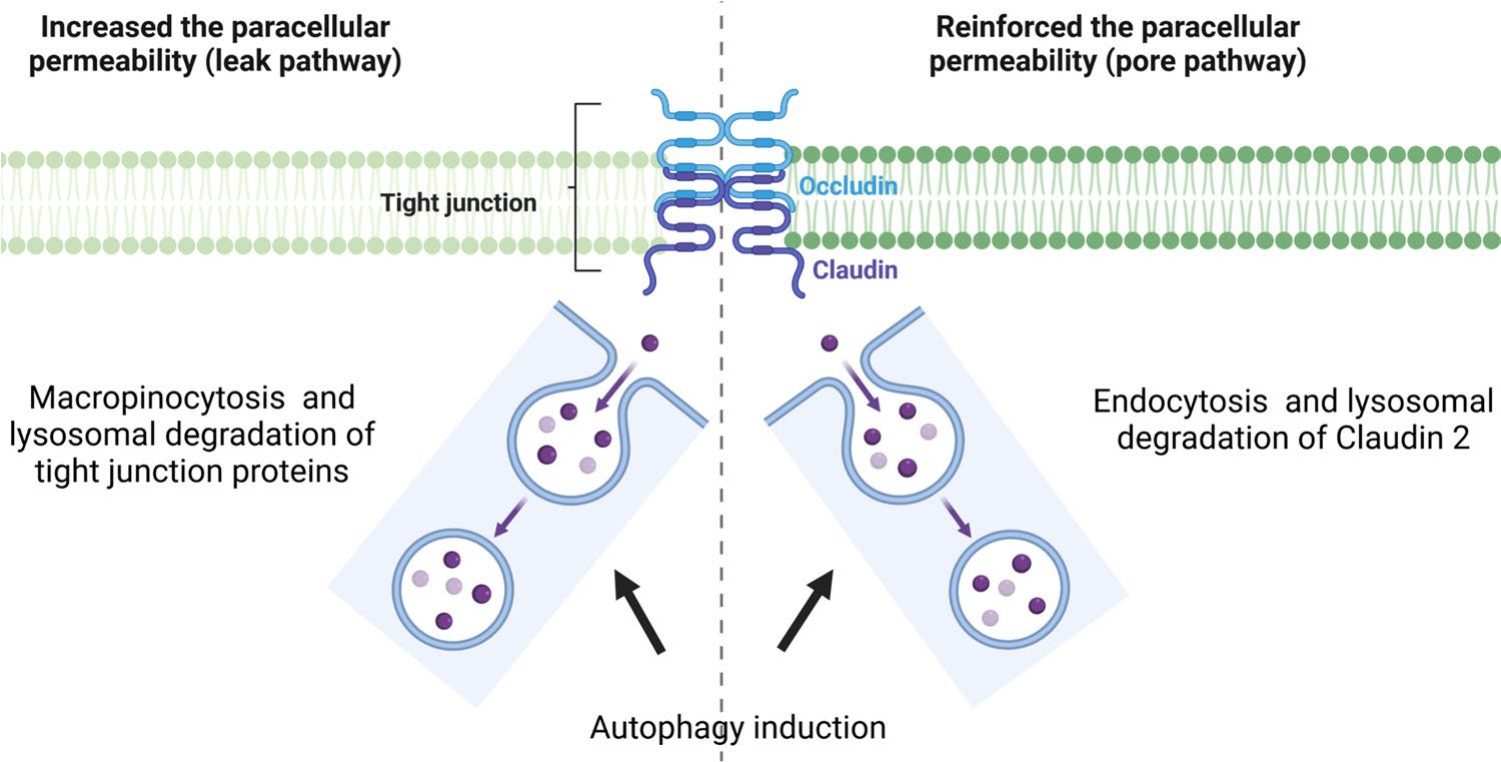
Schematic representation of the mechanisms induced by autophagy to control the intestinal permeability. Nowadays, scientific literature on the impact of autophagy on intestinal permeability is divergent. Autophagy induction has been shown to increase the paracellular permeability of intestinal epithelial cells from rodents and humans (left panel). This increased paracellular permeability, which is induced by autophagy, is due to the induction of macropinocytosis, which degrades the tight junction (TJ) proteins (left panel). In contrast, autophagy via lysosomal degradation of TJ protein claudin-2 (CLDN2), involving clathrin and adaptor protein AP2, reinforced the cohesion of Caco-2 monolayers by decreasing epithelial permeability (right panel)

Although the pivotal role of autophagy in the homeostasis of the gut mucosa is well described, very few studies have studied its impact on IP. Nevertheless, in addition to its deleterious impact on Paneth cells, food deprivation induced-autophagy, strongly increased IP as well as bacterial translocation to mesenteric lymph nodes (Fig. 4, left panel) [83]. The deleterious impact of autophagy on IP has been confirmed in Caco-2 monolayers as well as in mice models (Fig. 4, left panel).[84, 85] In more details, induction of autophagy by rapamycin or by nutrient starvation increased the paracellular permeability in Caco-2 cells and mice (Fig. 4, left panel).[85] Thus, increased paracellular permeability induced by autophagy is due to an induction of macropinocytosis degrading the TJ proteins (Fig. 4, left panel).[85, 86] In contrast, studies reported that autophagy via lysosomal degradation of the TJ protein Claudin 2 (CLD2) involving clathrin and adaptor protein AP2 reinforced the cohesion of the Caco-2 monolayers by decreasing epithelial permeability (Fig. 4, right panel) [87]. Moreover, the increased CLD2 level and TJ defects in Caco-2 monolayers treated with TNF-α partly arise from the inhibition of autophagy-mediated CLD2 degradation [88]. No modification of IP assessed in vivo was observed in knock-in rat model for the human *ATG16L1/T300A* as well as in knock-out rat for *Atg16l1*.[89] In contrast, IP was increased in *Atg16l1*^*ΔIEC*^ mice [90].

### IL-23 receptor, Th17 pathway immune Th17

Numerous reports have highlighted that numerous cytokines are involved in the development and perpetuation of IBD and IL23 in particular in CD pathogenesis [91, 92]. Thus, an increased level of IL23 have been described in the gut mucosa of CD patients [93]. IL23 is also mainly expressed by CD14^+^ intestinal macrophages, largely known to be involved in the perpetuation of inflammation by infiltrating into the inflamed intestine of CD patients [94]. Dendritic and epithelial cells have also been shown to produce IL23 [95]. Thus, a recent study has shown that mucosal TNFR2-expressing CD4^+^ T cells circumvent anti-TNF induced apoptosis by co-expressing IL23R, which is activated by the enhanced production of IL23 from mucosal macrophages, dendritic and epithelial cells [96]. Here, the activation of pSTAT3 in CD4^+^ mucosal T cells induced by IL23 promotes the resistance to apoptotic signals. These activated T cells are described to release high levels of inflammatory Th1 and Th17 (IL-17, IL-21, IL-22…) cytokines. These TNFR2^+^IL23R^+^T cells expand and accumulate in the mucosa of anti-TNF-refractory CD patients, where they perpetuate chronic inflammation [96]. These data imply that anti-TNF resistant patients could benefit from therapies specifically targeting IL23.

GWAS have analysed the polymorphism in the gene encoding IL23R and linked it to the CD pathogenesis, indicating the important role of IL23 in mucosal inflammation [92]. Especially the coding *R381Q* variant has been linked with functional consequences on T cell immunity [92]. Thus, CD patients carrying the protective variant of the IL23R produce reduced levels of IL17 and IL22 after IL23 stimulation, resulting in lower frequencies of circulating Th17 cells [97]. Moreover, these T cells display a reduced IL23 mediated phosphorylation of STAT3 and release less IL17 after stimulation by *Borrelia burgdorferi* [98]. Thus, these studies suggest that disease protective variants of the IL23R are more associated with reduced IL23R activity, whereas disease associated variants are more linked to elevated IL23R signaling.

**Fig. 5:**
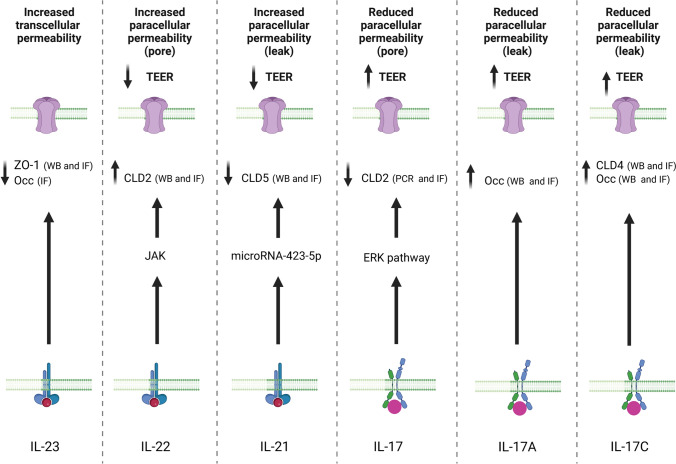
Graphical summary of the Th17 cytokines’ impact on the intestinal permeability. In the context of excessive Th17 immune induction, numerous Th17 cytokines are produced and released, including IL-23, IL-22, IL-21, IL-17, IL-17A and IL-17C. These cytokines have opposing effects on epithelial permeability. IL-23 increases transcellular permeability, while IL-22 and IL-17 regulate the pore pathway of paracellular permeability, and IL-21, IL-17A, and IL-17C control the leak pathway of paracellular permeability. Furthermore, although both IL-22 and IL-21 increase paracellular permeability, IL-17, IL-17A, and IL-17C reduce it. Specifically, IL-22 and IL-21 increase paracellular permeability by increasing Claudin 2 expression and reducing Claudin 5 expression, respectively. Conversely, IL-17, IL-17A, and IL-17C reduce paracellular permeability by decreasing Claudin 2 expression and increasing Occludin and Claudin 4 expression, respectively. *CLD2* Claudin 2, *CLD4* Claudin 4, *CLD5* Claudin 5, *ERK* extracellular signal-regulated kinase, *IF* Immuno-Fluorescence, *IL* Interleukin, *JAK* Janus Kinase, *Occ* Occludin, *TEER* Trans-epithelial electrical resistance, *WB* western Blot, *ZO-1* Zonula Occludens 1

Although the pivotal role of Th17 in gut homeostasis has been well documented, few studies have reported its impact on IP. A recent study has shown that small intestinal epithelial cell line (IEC-18) exposed to human breast milk with high level of IL-23 presented an increased transcellular flux of FD-70 (Fig. 5) [99]. ZO-1 protein expression, but not Occludin, was decreased by exposure to high levels of IL-23 [99]. Moreover, a reduced immunostaining of ZO-1 and Occludin were observed in epithelial monolayers exposed to breast milk with high IL-23 levels [99]. Thus, IL-23 in human breast milk is biologically active and increases IP through the degradation of TJ proteins (Fig. 5) [99]. Concerning the others cytokines of the Th17 immune profile (IL17, IL22 and IL26), it has been shown that basolateral stimulation of the respiratory epithelium by IL17, IL22 or IL-26 disrupted the epithelial barrier, evidenced by a reduced TEER, increased paracellular permeability of FD4, and discontinuous ZO-1 immunolocalization [100]. However, the first study reporting the impact of Th17 cytokine on IP has shown that IL17 stimulation increased the TEER and reduced the paracellular flux of mannitol across the T84 monolayers (Fig. 5) [101]. This reinforcement of the epithelial cell cohesion limiting the paracellular flux is mediated by ERK pathway activation, which reduces *CLD2* mRNA expression (Fig. 5) [101]. Moreover, in case of intestinal epithelial injury, IL17A has been described to play a beneficial role on the homeostasis of the epithelial monolayer by stimulating the expression of Occludin that limits the excessive permeability and maintains barrier integrity (Fig. 5) [102]. Similarly, IL17C is also described to maintain the IP by stimulating *Occludin* and *Claudin-4* mRNA expressions (Fig. 5) [103]. However, this positive impact on human epithelial monolayer has not been validated on Caco-2 cells, since IL17A treatment did not change Caco-2 TEER [104]. In contrast, IL17A has been recently shown to disrupt IP [105]. In vitro exposure of the intestinal epithelium to IL17A modified the TJ proteins’ localization including ZO-1, Claudin-5, and Occludin [105]. TJ disassembly was also accompanied by marked depolymerization of the peri-junctional F-actin cytoskeleton [105]. Finally, it has been shown that inhibition of IL-17 and IL-23 induced opposing effects in CD clinical trials [106]. Using a mouse model of IBD, Maxwell et al. have shown that IL17 inhibition weakened intestinal barrier and increased inflammation whereas IL-23 inhibition enhanced regulatory T cell accumulation, dampening inflammation [106]. Although IL21 and IL22 are critical for the maintenance of the intestinal barrier, their roles on IP have been poorly investigated [107–109]. Nevertheless, a recent study has evidenced that stimulation of IL21 altered the homeostasis of the Caco-2 monolayer by increasing the paracellular permeability (FD4 flux) and decreasing the TEER and mRNA expression of Claudin-5 (Fig. 5) [107]. Concerning the molecular mechanism, IL-21 altered the paracellular permeability by up-regulating the expression of microRNA-423-5p that reduced the mRNA and protein expression of Claudin-5 (Fig. 5) [107]. Concerning IL22, it has been shown that signals, exclusively through the basolateral side of Caco-2 cell monolayers, did not affect the flux of uncharged macromolecules across cell monolayers but significantly reduced TEER, indicating an increase of paracellular permeability for ions (Fig. 5) [108]. IL22 treatment on Caco-2 monolayers and on primary human intestinal epithelium markedly induced the expression of Claudin-2 (Fig. 5) [108, 109]. Furthermore, treatment of IL22 in mice upregulated CLD2 protein in colonic epithelial cells [108, 109]. IL22 mediated upregulation of CLD2 and loss of TEER can be suppressed by JAK inhibitors (Fig. 5) [108]. In humans, CLD2 and IL22 are both up-regulated in diseases with increased epithelial barrier permeability, like IBD [20].

### Vitamin D

There is growing evidence supporting that vitamin D could play an important role in IBD [110]. Several polymorphisms of the vitamin D receptors (VDR) have been studied for their effects in IBD, suggesting that they contribute to the variation in serum 25(OH)D levels in IBD patients [4]. The location of the VDR on chromosome 12, which is associated with IBD susceptibility [5], and the fact that VDR activation appears to be relatively weak in IBD patients compared with healthy controls, make genetic variants crucial variables affecting vitamin D status [111]. Finally, variants of the vitamin D binding protein open reading frame may influence IBD risk [112].

**Fig. 6:**
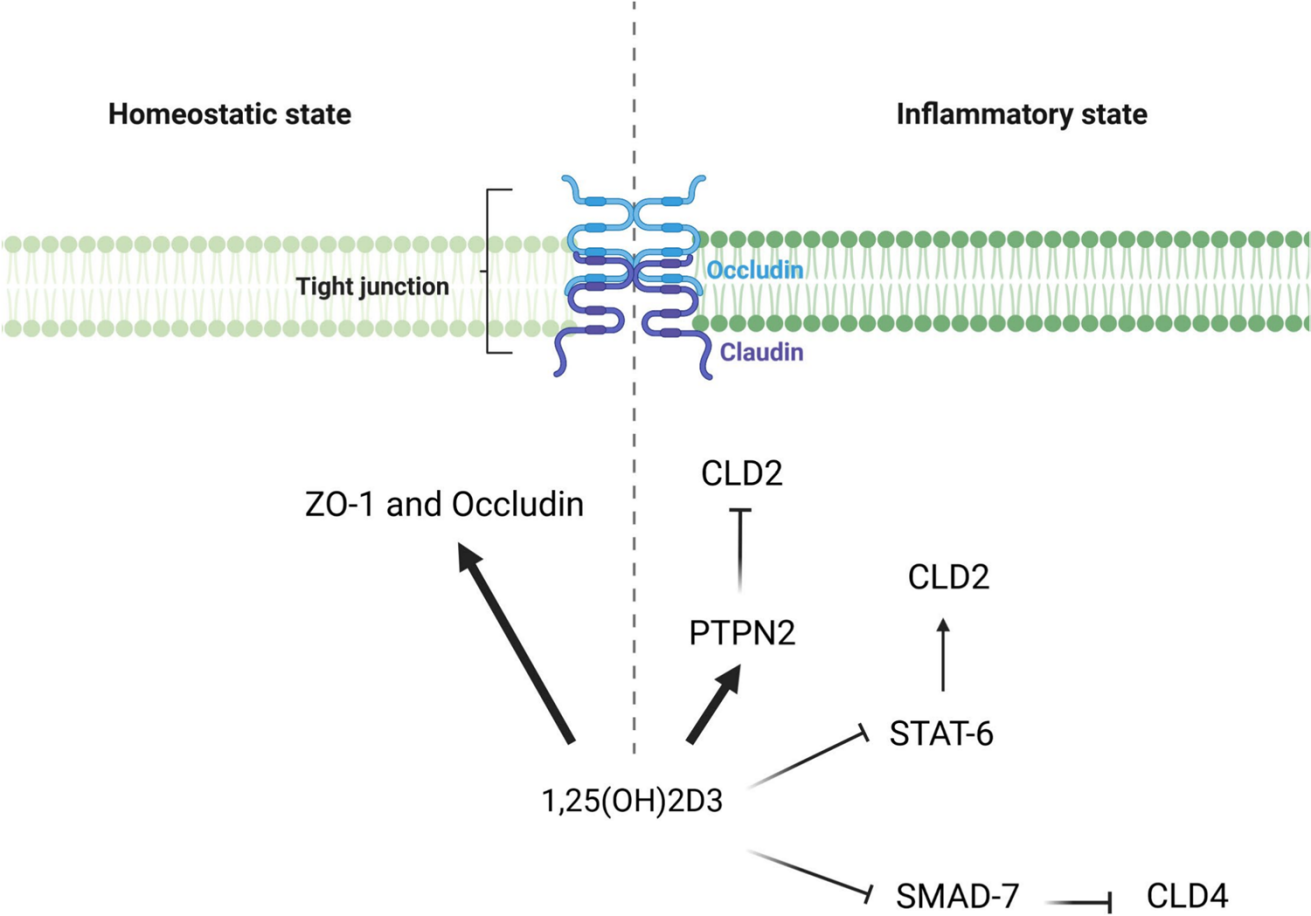
Graphical illustration of the mechanisms by which the vitamin D controls the intestinal permeability. In the homeostatic state (left panel), treatment with 1,25(OH)₂D₃ increases the expression of the tight junction (TJ) proteins ZO-1 and occludin. These proteins are known to reinforce the cohesion of TJ, thereby reducing paracellular flux. In the inflammatory state (right panel), which is characterised by a leaky epithelium, treatment with 1,25(OH)₂D₃ has been shown to reduce the expression of Claudin 2 (CLD2), which forms paracellular channels for small cations and water within the TJ. This reduced CLD2 expression is mediated by the ability of the 1,25(OH)₂D₃-VDR complex to enhance PTPN2 transcription, thereby limiting CLD2 expression (right panel). Furthermore, in patients with ulcerative colitis (UC), treatment with 1,25(OH)₂D₃ limits CLD2 expression by blocking STAT-6 phosphorylation and increases CLD4 expression (reducing pore size) by blocking SMAD-7 activity (right panel). *CLD2* Claudin 2, *CLD4* Claudin 4, *PTPN2* protein tyrosine phosphatase non-receptor type 2, *SMAD* *Caenorhabditis elegans* SMA (“small” worm phenotype) and MAD family (“Mothers Against Decapentaplegic”) of genes in *Drosophila*, *STAT6* Signal transducer and activator of transcription 6, *TJ* tight junction, *ZO-1* Zonula Occludens 1

The expression of TJ proteins, ZO-1 and Occludin, is induced in intestinal epithelial cells by treatment with 1,25(OH)2D3 (Fig. 6, left panel) [113, 114]. CLD2, is mainly expressed in leaky intestinal epithelium by forming paracellular channels for small cations and water within TJ [115]. CLD2 expression is induced by inflammatory cytokines, while it is repressed by tyrosine phosphatase N2 (PTPN2). Considering that the complex of 1,25(OH)2D3 and VDR promotes transcription of the PTPN2 gene, this could explain the inhibition of CLD2, which maintains the integrity of the intestinal epithelium (Fig. 6, right panel) [116, 117]. Moreover, CLD2 has been shown to be a target gene of VDR signaling even in the absence of inflammatory stimulation [118]. In the colon of UC patients, 1,25(OH)2D3 decreased CLD2 levels by blocking STAT-6 phosphorylation and increasing CLD4 levels by blocking SMAD-7 activity (Fig. 6, right panel) [119]. Moreover, overexpression of VDR in intestinal epithelia protects against colitis via TJ protein CLD15 upregulation [120].

## At the interface between the luminal content and the gut mucosal immune system, the sealing of the gut epithelium constitutes a promising therapeutic target for IBD

So far, most of the available treatment for IBD are immune-modulators aiming to dampen inflammatory response. This strategy has some risks while IBD patients under immunosuppressants and biologics are more susceptible to infections [121]. In this context, therapeutic strategies targeting epithelium healing without immunosuppression are attractive [122].

The digestive epithelium is a monolayer of epithelial cells that is essential for the intestinal homeostasis by regulating the crosstalk between the luminal content (dietary antigens, microbiota….) and the immune system. Thus, an excessive IP allows an increased passage of luminal contents across the epithelium then triggering abnormal stimulation of the immune system, which in turn alters the integrity of both gut epithelium and microbiota by secreting inflammatory mediators (cytokines, proteases…). Thus, in the context of Nod2 malfunction in hematopoietic cells following bone marrow transfer, the excessive secretion of inflammatory cytokines is able to alter IP without changes on the homeostasis of both mucus layer and gut microbiota [43, 56]. Moreover, in colitis context, Nod2 malfunctioning in hematopoietic cells is associated with an elevated colitis severity and the development of inflammatory lesions in the small intestine involving the re-circulating inflammatory CD4^+^ T cells [38, 55]. Reciprocally, according to the nature of the microbiota dysbiosis, this microbiota dysbiosis is able to trigger colonic inflammation [123], to enhance the severity of both colitis and the associated colorectal cancer lesions [45], and to alter the homeostasis of the mucus layer without changes in IP [124]. Thus, since the abnormal crosstalk between gut content and immune system promoted by an excessive IP plays critical roles in the genesis and/or relapses of IBD, the development of drugs targeting the regulation of the IP constitutes a new and promising therapeutic way.

Although advances have been done in understanding the mechanisms involved in the IP regulation, no molecule has presented efficiency to treat IBD by restoring IP homeostasis in clinical trials. For example, although the molecules blocking the MLCK activity have been described to be efficient to treat inflammation in mice models of colitis, none of these molecules have been efficient in clinical trials to treat IBD. Since the catalytic domain of MLCK is identical in epithelial, smooth, cardiac and skeletal muscle, targeting MLCK at the intestinal level will require alternative strategies. MLCK1 and 2 isoforms are specific to the intestinal epithelium [125]. Thus, MLCK1 increases IP and TNF enhances its expression and recruitment at the peri-junctional actomyosin site to alter the IP [125]. Moreover, The recruitment at the peri-junctional actomyosin site is dependent on IgCAM3 [126] and FKBP8 [127]. Two therapeutic strategies have been identified so far to remove MLCK1 from the peri-junctional actin-myosin meshwork and to improve IP: divertin (IgCAM3 target) and tacrolimus (FK506) (FKBP8 target).

Finally, no inhibitors of claudin-2 pore function are available to treat the excessive permeability. While a great deal has been learned about mechanisms of cell death, no agent able to prevent cell death to maintain barrier function have been studied in clinical trials. Thus, while a lot of work has been accomplished, further insight into mechanisms of the disease and the development of novel therapeutic agents are needed before direct therapy of intestinal barrier function can be considered seriously.

## Conclusions

Intestinal permeability is an important feature of the gut epithelial barrier, since many essential nutrients are absorbed at this interface, along with normal microbiota-priming of the gut-associated immune system via the passage of antigens. However, excessive permeability favours and/or exacerbates chronic inflammation, leading to debilitating diseases. By presenting the impact of the main inflammatory pathways involved in IBD on the intestinal permeability, this review has highlighted the urgent need to develop new molecules targeting the intestinal epithelium to correct the permeability default favouring the excessive passage of luminal components overstimulating the immune system leading to chronical inflammation.

## Abbreviations

AGR2: Anterior gradient 2
AMP: Antimicrobial peptides
ATF6: Activating transcription factor 6
*ATG16L*: *Autophagy* Related 16 like gene
CD: Crohn’s disease
CHOP: CCAAT-enhancer-binding protein homologous protein
CLD: 2Claudin 2
eIF2a: Eukaryotic Translation Initiation Factor 2A
ER: Endoplasmic reticulum
FD: Fitc-Dextran
GRP7: 8Glucose regulated protein 78
GWAS: Genome-wide association study
HRP: Horseradish peroxidase
IBD: Inflammatory Bowel Disease
IEC: Intestinal epithelial cells
IFN: Interferon
IL: Interleukin
IP: Intestinal permeability
IRE1: Inositol-requiring protein 1()
IRGM: Immunity-related GTPase family M
MAPK: Mitogen activated protein kinase
MDP: Muramyl dipeptide
MLCK: Myosin light chain kinase
NF-κB: Nuclear factor-κB
NOD2: Nucleotide-binding oligomerization domain 2
PERK: Protein kinase RNA (PKR)-like ER kinase
PGN: Peptidoglycan
PP: Peyer patches
TEER: Trans-epithelial electrical resistance
TJ: Tight junction
TLR: Toll like receptor
TNF: Tumor necrosis factor
UC: Ulcerative Colitis
UPR: Unfolded protein response
VDR: Vitamin D receptors
XBP-1: X-Box Binding Protein
